# The influence of targeted therapies in the inner ear

**DOI:** 10.3389/fphar.2025.1620679

**Published:** 2025-09-17

**Authors:** Tony Bonilla, Jake DiFatta, Esperanza Bas Infante, Stefania Goncalves

**Affiliations:** ^1^ Neuroscience Program, Howard College of Arts and Sciences, Samford University, Homewood, AL, United States; ^2^ Department of Otolaryngology—Head and Neck Surgery, Heersink School of Medicine, The University of Alabama at Birmingham, Birmingham, AL, United States; ^3^ Investigational Drug Services, University of Miami, Coral Gables, FL, United States

**Keywords:** ototoxicity, targeted therapies, monoclonal antibodies, hearing loss, small molecules

## Abstract

Scientific research has significantly propelled advancements in healthcare. One notable application is precision medicine, which seeks to analyze and comprehend disease pathology to offer personalized medical treatments to patients. Targeted oncology, a branch of precision medicine, focuses on identifying and targeting specific molecules that regulate cancer cells, thereby minimizing harm to healthy cells. Different types of targeted therapy against cancer include monoclonal antibodies and small molecules. This manuscript intends to provide an overview of the influence of these targeted oncology and non-oncology therapies on hearing. Furthermore, side effects including immune-related adverse events will be reviewed as potential causes of hearing deterioration in this patient population.

## Introduction

Pharmacologic therapies intended to treat malignancies also affect normal cells, leading to significant peripheral neuropathy sequelae including ototoxicity (e.g. hearing loss, tinnitus, dizziness, and/or vertigo) (Landier, 2016). The long-term sequelae of this can be devastating, as it can lead to bilateral severe to profound sensorineural hearing loss and subsequent developmental delay and poor communication skills in children, (Tomblin et al., 2015), as well as social isolation and higher risk of dementia in adults (Chern and Golub, 2019; Brewster et al., 2022). The prevalence of chemotherapy-induced ototoxicity is alarming, with reported incidence rates higher than 50% (Kessler et al., 2024) and a prevalence that ranges from 4% to 90% (Schell et al., 1989; Knight et al., 2005; Gupta et al., 2006; Dean et al., 2008; Lewis et al., 2009; Nitz et al., 2013; Landier et al., 2014). Among all available therapeutic options, platinum-based agents are known to be the most ototoxic, affecting more than half a million patients annually in the United States alone (Travis et al., 2014; Dillard et al., 2022). There are several factors that can increase a patient’s risk for developing ototoxicity, including age, chemotherapeutic agent used, cumulative dose, infusion rate, and genetic predisposition (Landier, 2016; Kessler et al., 2024).

The concept of precision medicine or ‘personalized medicine’ has revolutionized the prevention and treatment of many diseases. In oncology, it emphasizes treatment customization based on the distinct biological characteristics of tumors, optimizing therapeutic effects against malignant cells while minimizing side effects that result from damage to healthy cells and organs (Tsimberidou et al., 2020). Over recent years, targeted immunotherapies have rapidly developed, becoming essential components in the treatment of many cancers and other conditions (Shahid et al., 2019). The role of immunotherapy in cancer treatment is rapidly evolving; in some malignancies, it serves as a supplement to traditional chemotherapy or radiation, while in diseases such as melanoma, the use of monoclonal antibody (mAb) monotherapy has become the gold-standard (Choi et al., 2020; Zhang et al., 2024). Understanding how these targeted therapies are integrated with conventional treatment is essential when interpreting reports of hearing loss. A case of ototoxicity in a patient receiving combination therapy (chemotherapy and/or radiation plus a mAb) is markedly different in significance when compared to a patient treated with mAb or small molecule monotherapy who develops hearing loss. The causal link is potentially more direct in the latter case.

Two main categories of immunotherapy include mAbs and small molecules. Monoclonal antibodies function by targeting and binding to specific antigens, such as those presented by cancer cells or microbes, to enhance the immune system recognition and response. These drugs operate through various mechanisms, including activating/blocking signaling cascades and promoting opsonization (Shahid et al., 2019; Zhang and Zhang, 2020). Small molecules similarly target specific biochemical pathways to affect cancer cell growth and proliferation via competitive inhibition of enzymes, targeting oncogenic pathways to induce apoptosis and block angiogenesis (Zhong et al., 2021; Chattopadhyay et al., 2024; Li et al., 2024). While side effects and adverse effects of these targeted therapies have been described and are routinely monitored, their ototoxic and vestibulotoxic effects may be underestimated. These therapies have been sparingly reported on and their ototoxic effects have been difficult to quantify due to an absence of protocol-based screening (Naples et al., 2023). It is hypothesized that damage to the inner ear associated with these drugs occurs through previously described immune pathways categorized as immune-related adverse events (irAEs) (Rosner et al., 2020).

Immune-related ototoxicity from immunotherapeutics may arise through several mechanisms. First, mAbs can cause direct complement-mediated tissue injury, as seen in myocarditis cases with elevated PD-L1 expression, suggesting that similar off-target effects could occur in the inner ear (Engelhardt et al., 2020). Second, these drugs may promote autoantibody production by enhancing T and B cell interactions, leading to immune attacks on cochlear structures in susceptible individuals (Das et al., 2018). Another possible mechanism is depletion of regulatory T cells and promotion of Th17-driven cytokine release, including IL-17 and TNF-α, which are implicated in autoimmune disease and may mediate inner ear inflammation (Gianchecchi and Fierabracci, 2018; Yang et al., 2018).

A different category of immunotherapy is the use of engineered T cells, including chimeric antigen receptor (CAR) T cells, T cell receptor (TCR) modified lymphocytes and tumor-infiltrating lymphocytes (TILs), which have been associated to a few cases of hearing loss, especially in patients suffering from melanoma (Seaman et al., 2012; Duinkerken et al., 2019). While this type of cellular therapy is not discussed in this manuscript, it is worth noting that its impact on hearing loss may be due to primed immune cells targeting healthy melanocyte-like cells within the stria vascularis of the inner ear (Barozzi et al., 2015).

This manuscript reviews targeted oncology therapies as a subset of precision medicine, focusing on the existing evidence of ototoxicity and vestibulotoxicity in mAb and small molecule therapies.

## Methods

A thematic review was conducted to examine reported cases of hearing loss, ototoxicity, and vestibulotoxicity associated with mAbs and small molecule therapies. A comprehensive literature search was performed using PubMed for all relevant articles available through April 2025, without restriction on earliest publication date. Search keywords included the name of the monoclonal antibody or small molecule, combined with terms including “ototoxicity,” “hearing loss,” or “vestibulotoxicity.” The search focused on studies reporting auditory and/or vestibular adverse effects related to systemic administration of these agents. Eligible sources included systematic analyses, database analyses, and case reports, all of which were screened for relevance to otologic or vestibular outcomes. Where available, data were extracted on the number of patients or treatments, duration of therapy, and audiometric findings both before and after an event, including pure tone audiometry results. Studies were excluded if they lacked specific mention of auditory or vestibular symptoms, if adverse effects occurred prior to the administration of the targeted therapy, or if full-text access was unavailable in English.

## Monoclonal antibodies (mAb)

mAb therapies, particularly those targeting immune checkpoints and specific cancer markers, have been associated with ototoxicity and vestibulotoxicity in rare instances. These toxicities are generally considered immune-mediated, often arising from the immune system’s activation or dysregulation, leading to inflammation of the inner ear structures (Rosner et al., 2020) causing intracochlear vasculitis or cross-reactivity with hair cells (McKeage and Perry, 2002; Belinsky et al., 2022). To date, ototoxicity and/or vestibulotoxicity associated with mAb treatment has been described in a few dozen case reports (Cheminant et al., 2012; Charakopoulos et al., 2020; Nocturne et al., 2021) and a small number of systematic reviews (Arya et al., 2025). Inner ear toxicity is not a common side effect of monoclonal antibodies, and when it does occur, it is often reversible with appropriate management, primarily involving corticosteroids. These side effects remain rare but underscore the importance of early monitoring and prompt intervention (Thompson et al., 2024; Arya et al., 2025).

### Monoclonal antibodies in oncology treatments

#### Programmed Death-1 (PD-1)

Pembrolizumab and nivolumab are both PD-1 inhibitors and were first approved by the FDA in 2014 for the treatment of metastatic melanoma (Gong et al., 2018). Both are now used widely for many other cancers and are often used as dual therapy with ipilimumab, a Cytotoxic T-Lymphocyte-Associated Protein 4 (CTLA-4) inhibitor, due to their synergistic effects in targeting tumor cells (Wei et al., 2018). Sudden hearing loss, tinnitus, and aural fullness have been described in cases where these agents were used both alone and in combination therapy. For all three agents, the development of an autoimmune response against healthy host melanocytes in the inner ear has been proposed as a mechanism of toxicity (Rosner et al., 2020).

Kuzucu et al., 2019, used a rat model to investigate the ototoxic effects of pembrolizumab, reporting that the drug showed ototoxicity activity during treatment with spontaneous resolution after completion during the follow up assessment. This study drew conclusions from auditory brainstem response measurements and histological evidence of insult to the organ of Corti, but did not propose a mechanism of injury (Kuzucu et al., 2019). These findings have been supported by several other publications reporting this phenomenon in humans. Wierzbicka et al., 2024, performed a systematic review noticing that patients developed ototoxicity symptoms about 3 months into the initiation of treatment, with most cases being reversible. Little is known regarding the true incidence and prevalence of this problem. They suggested the creation and implementation of therapeutic algorithms to allow for early screening, diagnosis, and management of symptoms (Wierzbicka et al., 2024). Noteworthy, Page and collaborators published a case series from MD Anderson and a systematic review where four patients presented with inner ear toxicity (Page et al., 2022). Three patients were treated with nivolumab combined with ipilimumab for metastatic melanoma and renal cell carcinoma, while the remaining patients received combined therapy for the treatment of metastatic melanoma. All patients underwent treatment of hearing loss with high dose steroid tapers, leading to recovery of hearing in 2 patients, persistent symptoms in one, and cancer-related death in one. Schlacter et al. described a case of melanoma treated with combination therapy (ipilimumab and nivolumab) leading to bilateral profound hearing loss that did not resolve with systemic steroid therapy, requiring cochlear implantation surgery (Schlacter et al., 2023). By the time of this publication, only 13 audiovestibular adverse effects have been implicated as immune-related adverse events (irAEs), highlighting that indeed, it is a rare complication.

#### Programmed death ligand – 1 (PD-L1)

Durvalumab is a PD-L1 inhibitor that is FDA-approved for the treatment of non-small cell lung cancer (NSCLC), extensive-stage small cell lung cancer (SCLC), locally advanced or metastatic urothelial carcinoma, advanced or metastatic biliary tract cancer, hepatocellular carcinoma, endometrial cancer, and as a neoadjuvant therapy for resectable NSCLC (Wilmington, 2024). As per the manufacturer disclosure, Durvalumab has been associated with an 11-20% rate of dizziness in patients undergoing treatment for HER2-Negative, high risk early breast cancer, metastatic castration-resistant prostate cancer, and BRCAm advanced ovarian cancer (Yang et al., 2018). Of note, most of these patients were concurrently or previously treated with other chemotherapeutic agents that could potentially be ototoxic, therefore, a direct relationship cannot be confirmed.

One case series described irAEs in four out of seventeen patients, including sudden hearing loss in two patients and tinnitus in one patient receiving durvalumab and Olaparib, a poly (ADP-ribose) polymerase inhibitor (PARP), in the metastatic castration treatment of resistant prostate cancer (Karzai et al., 2018). Patients received a median of seven cycles of treatment, and all patients experiencing an irAE discontinued durvalumab but not Olaparib. Neither patient received audiometric testing, and both were treated with high-dose steroids. One patient was reported to have subjective near-recovery of hearing loss, while the other patient required hearing aids. Low-grade tinnitus was documented in one patient, but it is unclear whether this occurred in a patient who experienced hearing loss or a different, third patient (Karzai et al., 2018).

Interestingly, De Boos and collaborators (De Boos et al., 2025), published a case report of a 59-year-old man with non-small cell lung carcinoma who underwent concurrent chemoradiation (cisplatin-pemetrexed) followed by immunotherapy with durvalumab immunotherapy. Despite achieving a complete metabolic response on PET-CT, he developed solitary metastasis to the internal auditory canal 18 months later, experiencing rapid progressive vertigo, left-sided facial paralysis, and hearing loss. It is difficult to draw conclusions from this case as the patient was treated with a known ototoxic drug, cisplatin, within the same timeframe prior to hearing loss in addition to later developing retrocochlear metastatic disease that can also lead to audiovestibular symptoms.

#### CD20

Rituximab depletes B cells by targeting CD20. It is used in the treatment of autoimmune conditions like rheumatoid arthritis, microscopic polyangiitis, granulomatosis with polyangiitis, pemphigus vulgaris, and B cells malignancies like CD20-positive B-cell non-Hodgkin’s Lymphoma, chronic lymphocytic leukemia, and small lymphocytic leukemia (Hanif and Anwer, 2025). The off-label use of this medication has expanded from demyelinating diseases to hematologic, oncologic, vascular, and dermatologic conditions and others, increasing its use from 1.2% in 2009 to over 50% by 2017 (Delate et al., 2020).

There is no proposed mechanism by which rituximab or B-cell depletion broadly contributes to hearing loss, and no animal studies assessing the effect of rituximab on the inner ear were found. Across two country-wide pharmacovigilance studies within the FDA Adverse Event Reporting System (FAERS) database measured reporting odds ratios (ROR). ROR are commonly used in pharmacologic database surveys to indicate disproportionate drug-specific reporting of an adverse effect versus all other drugs in a given database. Statistically significant reporting ROR of ∼3.20 has been measured for hearing loss (Barbieri et al., 2019). Two case reports have described a total of three patients developing progressive hearing loss, with two experiencing balance/gait disturbance while on rituximab maintenance therapy following remission of B-cell lymphoma. In all three cases, patients experienced systemic enterovirus infection due to presumed rituximab-associated hypogammaglobulinemia (Anderson et al., 2022). One report describes subtle sensorineural hearing confirmed with audiometry after 4 months of maintenance single-therapy (Healy et al., 2015). The patient presented with balance impairment and progressive hearing loss was treated with intravenous immunoglobulin (IVIG) and steroids over 20 weeks. Despite treatment, the hearing function precipitously worsened, requiring cochlear implantation surgery. Similarly, the other two patients did not respond to multiple weeks of IVIG therapy and experienced persistent hearing loss, one of which also experienced gait disturbance that resolved (Grisariu et al., 2017).

Ocrelizumab is a mAb against CD20 approved for the treatment of multiple sclerosis. Starosta et al. present a case in which a patient diagnosed with multiple sclerosis contracted a systemic enterovirus infection that resulted in sensorineural hearing loss along with hepatitis, pneumonia, enterocolitis, and pancreatitis (Starosta et al., 2025).

Viral infections are considered an important underlaying cause of sensorineural hearing loss and have also been associated to enterovirus infection (Belinsky et al., 2022). Therefore, the reported hearing loss published by Anderson and Starosta is likely post-viral in nature, rather than due to the use of rituximab or ocrelizumab.

### Monoclonal antibodies in non-oncology treatments

#### Insulin-like growth factor (IGF)

Teprotumumab is an IGF-1R inhibitor indicated in the treatment of thyroid eye disease (TED). It received FDA approval in January 2020 but has not comprehensively been evaluated from a safety perspective in the real-world clinical setting (Yvon et al., 2025). The IGF-1R pathway has been described as a component of cochlear maturation and regulation (García-Mato et al., 2021), which may help explain the ototoxic symptoms that have been reported (Najjar and Yu, 2022). One pharmacovigilance analysis of teprotumumab reported significant ROR signals for development of autophony (ROR = 14,475.49), permanent deafness (ROR = 1853.35), unilateral sensorineural hearing loss (ROR = 129.89), ear discomfort (ROR = 72.88), and bilateral sensorineural hearing loss (ROR = 62.46), among other non-otologic effects (Zhang et al., 2024), which have been supported by other studies using a similar analytic approach (Huynh et al., 2024; Zhao and Tao, 2024). Other case studies have reported sensorineural hearing loss, tinnitus and autophony in 10%–46% of patients undergoing treatment for TED (Keen et al., 2024) and ear fullness to secondary atrophy of the tissues surrounding the Eustachian tube valve (Hsiou et al., 2024). The effects of IGF-1R deficiency have been well-described in both mouse models and via identification of relevant human genes. Studies have demonstrated that the IGF-1R signaling pathway plays a critical role in cochlear development, homeostasis, and protection, with tightly regulated expression patterns and downstream effectors whose dysfunction is linked to various forms of hearing loss and potential therapeutic targets (Murillo-Cuesta et al., 2011; Okano et al., 2011; García-Mato et al., 2021).

#### CD3

Muronumab is an anti-CD3 mAb that blocks the cytotoxic activity of T cells, therefore, it is used in the treatment of transplant-related rejection that has been resistant to conventional therapy (Todd and Brogden, 1989). Hartnick and collaborators (Hartnick et al., 1997) reported associated temporary sensorineural hearing loss confirmed by audiogram in one case study. The patient was undergoing treatment alongside renal transplantation, and experienced sudden hearing loss upon administration of the first dose. Audiograms showed bilateral, down-sloping sensorineural hearing loss with discriminations of 92% and 88%, respectively. The patient responded to steroids and experienced subjective restoration of hearing function. The proposed mechanism by which the hearing loss occurred is significant cytokine release, altering the vascular permeability of the organ of Corti. In 2000, the same group presented a case series of 7 patients that underwent therapy with muronomab due to steroid-resistant rejection of renal cadaveric transplants reporting 71% of sensorineural hearing loss of at least 15 dB at high frequencies. All patients experienced near-complete to complete resolution of the hearing loss up to 2 weeks after discontinuation of the drug (Hartnick et al., 1997). The authors propose that muronumab may damage the inner ear through a “first-dose” immune response, characterized by an unusually large release of cytokines in the organ of Corti or surrounding tissues, leading to vasodilation and inflammation (Hartnick et al., 1997).

#### Tumor necrosis factor–Alpha (TNF- α)

The role of TNF- α as an inflammatory mediator contributing to hearing loss has been well established, with multiple proposed mechanisms linked to hearing loss. TNF- α infusion led to direct synaptic degradation of the cochlear nerve, subsequently reversed by administration of etanercept in an animal model (Kessler et al., 2024). TNF- α has further been reported to decrease cochlear blood flow (Schlacter et al., 2023) that was reversed by systemic etanercept infusion (Shahid et al., 2016), a soluble TNF receptor that binds and blocks TNF signaling. The described pathways complicate the significance of reports of hearing loss linked to TNF- α inhibitor use and must be acknowledged.

Adalimumab has been reported in the treatment of autoimmune sensorineural hearing loss, however, Conaway et al., 2011, reported 2 cases of sensorineural hearing loss related to the use of Adalimumab and calls for its cautionary use in this setting (Conway and Khan, 2011). Both patients started using Adalimumab after intolerance to Methotrexate and experienced unilateral sensorineural hearing loss after several months of use. These patients were evaluated by an otolaryngologist and underwent appropriate work up with audiogram and MRI.

Etanercept and infliximab have been associated with hearing loss especially when combined with methotrexate or when used for extended periods of time. In a study published by Savastano (Savastano et al., 2010), 28 patients with ankylosing spondylitis were treated with either drug alone or in combination with methotrexate. Patients were followed up with audiometric testing showing that 57% of patients were diagnosed with sensorineural hearing loss. All patients that used combination therapy developed sensorineural hearing loss whereas only 43% of those on monotherapy experienced decreased hearing. Patients with known exposure to other ototoxic drugs, noise, Meniere’s disease, head trauma and metabolic diseases, were excluded. This study did not describe the timeline of audiologic testing, if a baseline hearing test was performed before initiation therapy, and if all the patients underwent testing or not, limiting the extrapolation of its conclusions.

In a two-patient case report of patients with Cogan’s syndrome, one subject experienced sensorineural hearing loss while on infliximab and one patient experienced hearing improvement, while the other experienced vertigo and hearing loss, confirmed by pure-tone audiometry (Touma et al., 2007).

While the protective effects of TNF- α inhibitors against cochlear damage and SNHL have been described, there may be more that we are yet to understand regarding the long-term use of these drugs, especially alongside the use of other immune-modulating agents such as methotrexate. The mechanism of action of these drugs leading to inner ear ototoxicity has not been described (Moore et al., 2023).

Some limitations of the above-mentioned studies are: the small sample size that limits the establishment of a cause-effect relationship and the confounding factor of the presence of an active autoimmune disease that could manifest with sudden sensorineural hearing loss when the disease is not in remission. Therefore, further studies are needed to determine a real association.

##### Other monoclonal antibodies (Mab)

Sudden hearing loss has been reported as an unexpected safety signal in a FAERS database scrape for daratumumab, a CD38-targeting mAb used to treat multiple myeloma. However, the study does not report the specific risk for hearing loss, and it is likely that the signal intensity is statistically insignificant (Yun et al., 2024). Further, database surveys are unable to establish causality or qualified risk. No mechanism of toxicity has been proposed to date, and no case reports have been located describing describe inner ear toxicity with its use.

Erenumab and fremanezumab, both approved by the FDA in 2018, block the calcitonin gene-related peptide (CGRP) receptor to prevent migraine in adults. Hearing loss has been described in one patient who was treated for chronic migraine with erenumab for 6 months before switching to fremanezumab. While taking fremanezumab, the patient presented with unilateral hearing loss. They ultimately underwent mastoidectomy and paranasal biopsy, which led to a diagnosis of granulomatosis with polyangiitis (GPA) (Ray et al., 2021). The causality of either mAb leading to the unilateral hearing loss is unable to be established in this case, as GPA can lead to either sensorineural or conductive hearing loss on its own. The mechanism of action leading to changes in hearing is hypothesized to be chronic nasopharynx inflammation may cause Eustachian tube dysfunction and subsequent chronic serous otitis media presenting with conductive hearing loss. On the other hand, vasculitis of the inner ear can cause ischemia of the cochlea or auditory nerve leading to sensorineural hearing loss (Mur et al., 2019; Cacco et al., 2021; Busch et al., 2022). CGRP has been hypothesized to cause hearing loss via vasodilatory effects and increased recruitment of leukocytes to the middle and inner ear, though the authors note that the possible association drawn is speculative and not bolstered by a known immunochemical pathway (Ray et al., 2021).

Denosumab is a RANK-L inhibitor used in the treatment of osteoporosis and several bone-related cancers to prevent osteoclast activation and bone resorption. To date, denosumab has been described in one case of bilateral external auditory canal osteonecrosis in a 79-year-old woman who presented with symptoms of sudden hearing loss and otalgia 4 months after treatment initiation (True et al., 2021). This patient had been taking once-daily bisphosphonates for over 10 years for the treatment of osteoporosis prior to switching to denosumab. The patient was treated conservatively over the course of 1 year with an initial treatment of a 7 day, three-times-daily course of combined neomycin/dexamethasone/acetic acid ear spray, with ongoing aural toilet including six microsuction sessions, removal of bone debris and mastoid mucoid discharge, and regularly refreshed wick dressings with ointment. At 1 year follow-up, the patient reported no otalgia or hearing loss. The patient did not undergo formal audiometric assessment to quantify the degree of initial hearing loss or subsequent recovery. Although no studies were found describing a mechanism by which RANK-L inhibition may cause the symptoms described, it is possible that the inhibition of the RANK/NF-κB pathway may have played a paradoxical effect on the inflammatory response in this patient.

Trastuzumab, first approved in 1998, is a HER-2 antagonist primarily used in the treatment of HER-2 positive breast and gastric cancers. No evidence suggests that trastuzumab has significant inner ear effects in humans. One laboratory animal study in rodents showed that HER-2, a transmembrane receptor tyrosine kinase, can be found in the inner, suggesting a hypothetical mechanism of damage or inflammation (Eryilmaz et al., 2016). However, one pharmacologic adverse event database survey did not find a statistically significant relationship between trastuzumab and hearing loss (Favrelière et al., 2020). Table 1 provides a summary of the discussed monoclonal antibodies.

**TABLE 1:** Summary of monoclonal antibodies and inner ear effects reported.

Monoclonal antibody	Drug classification	Hearing effects reported	References
Teprotumumab	IGF-1R Inhibitor	- Bilateral mild/moderate/severe SNHL, tinnitus, popping, autophony	Belinsky et al. (2022) Chow and Silkiss (2022) Douglas et al. (2023) Ding et al. (2022) Highland et al. (2022) Kahaly et al. (2021) Kay-Rivest et al. (2023) Keen et al. (2024) Lu et al. (2023) Men et al. (2024) Najjar and Yu (2022) Phansalkar et al. (2023) Reed et al. (2022) Sears et al. (2022) Shah et al. (2024) Yu et al. (2021)
Pembrolizumab	PD-1 Inhibitor	- Bilateral mild/moderate/severe SNHL, tinnitus, vertigo, aural fullness	De Groot et al. (2020) Hobelmann K, Fitzgerald D (2019) Page et al. (2022) Rosner et al. (2020) Stürmer et al. (2021)
Nivolumab	PD-1 Inhibitor	- Bilateral mild/moderate/severe SNHL, tinnitus, vertigo, aural fullness, imbalance	Choi et al. (2020) Gambichler et al. (2020) Lemasson et al. (2019) Nagai et al. (2023) Page et al. (2022) Rajapakse et al. (2020) Rosner et al. (2020) Schlacter et al. (2023) Stürmer et al. (2021) Tampio et al. (2021)
Ipilimumab	CTLA-4 Inhibitor	- Bilateral mild/moderate/severe SNHL, tinnitus, vertigo, aural fullness, imbalance	Choi et al. (2020) Monferrer-Adsuara et al. (2021) Page et al. (2022) Rosner et al. (2020) Schlacter et al. (2023) Stürmer et al. (2021) Voskens et al. (2012)
Durvalumab	PDL-1 Inhibitor	- Bilateral moderate to severe SNHL, unilateral CHL, mild tinnitus, vertigo	Karzai et al. (2018) De Boos et al. (2025)
Rituximab	Anti-CD20	- Bilateral mild/moderate SNHL, gait disturbance/vertigo, labyrinthine damage, hypoacusis	Barbieri et al. (2019) Favreliere et al. (2020) Grisariu et al. (2017) Healy et al. (2015)
Muromonab	Anti-CD3	- Bilateral mild SNHL	Hartnick et al., 1997
Adalimumab	TNF-<Inhibitor	- Unilateral SNHL, bilateral SNHL, tinnitus	Barbieri et al. (2019) Conway et al. (2011) Favreliere et al. (2020)
Etanercept	TNF-<Inhibitor	- Bilateral moderate/severe SNHL, tinnitus	Savastano et al. (2010)
Infliximab	TNF-<Inhibitor	- Bilateral severe SNHL, tinnitus, both worsened and improved vertigo and hearing (different patients)	Barbieri et al. (2019) Savastano et al. (2010) Touma et al. (2007)
Daratumumab	Anti-CD38	- FAERS database safety signal for sudden hearing loss, ear and labyrinth disorders	Yun et al. (2024)
Erenumab	Anti-CGRP	- Unilateral CHL vs SNHL iso GPA	Ray et al. (2021)
Fremanezumab	Anti-CGRP	- Unilateral CHL vs SNHL iso GPA	Ray et al. (2021)
Denosumab	RANK-L Inhibitor	- Bilateral moderate/severe CHL	True et al. (2021)

### Small molecules

To date, there are over 80 small molecules approved by the FDA for the treatment of cancer (Zhong et al., 2021). Even when these drugs have some of the advantages of mAbs, these still face many challenges including low response rate and potential development of drug resistance. Small molecules can have different targets such as kinases, kinase receptors, regulatory proteins, proteosomes, and DNA damage repair enzymes (Zhong et al., 2021).

#### Tyrosine kinase inhibitors (TKI): imatinib, gefitinib, erlotinib, sunitinib

Imatinib was the first small molecule TKI approved for use. Initially approved in 2001 for use in Chronic Myeloid Leukemia (CML), it has since been approved for use for Gastrointestinal stromal tumors (GISTs) in 2003 and acute lymphoblastic leukemia (ALL) in 2006. The primary targets include BCR-ABL, PDGFR-β, and c-kit tyrosine kinases (Flynn and Gerriets, 2025). One single center, cross sectional study from northern India reported 4 cases of imatinib-induced ototoxicity, implicating imatinib in the development of bilateral sensorineural hearing loss and tinnitus with normal caloric testing. Another study reported 44 patients treated with 400 mg oral imatinib per day who developed hearing loss within 6 months of initial treatment (Gupta et al., 2017). Lastly, one case reported the development of symptoms over 1 year after initial treatment with multiple interruptions during therapy due to resection and recurrence of a GIST and the development of other adverse effects such as mouth sores, facial swelling, and lower extremity rash (Wasif et al., 2016). All cases reported no improvement in hearing after otologic symptoms occurred.

Erlotinib is an epidermal growth factor receptor (EGFR) tyrosine kinase inhibitor that has been approved for use in NSCLC in 2004 and pancreatic cancer in 2005 (Carter and Tadi, 2025). There has only been one case report of erlotinib-induced ototoxicity, in a patient undergoing treatment for pancreatic adenocarcinoma. This 66-year-old gentleman started on 150 mg of erlotinib daily, and 30 min following the first dose, he experienced sudden-onset tinnitus, aural fullness, and severe asymmetric sensorineural hearing loss. An audiogram later showed complete deafness of the right ear and severe SNHL in the left. The patient died several weeks after the audiogram with no improvement in hearing. The proposed mechanism of action is that the expression of EGFR has been shown in sensory and non-sensory cells in the inner ear and is potentially associated with the survival and proliferation of cells and synaptic maintenance. Therefore, inhibition of this receptor could affect the homeostasis of the inner ear by decreasing inner ear cell survival and affecting synapsis (Koutras et al., 2008).

Gefitinib is another EGFR TKI that was approved for use in NSCLC in 2003 (Cohen et al., 2003). There has only been one case associated with ototoxic side effects. An 81-year-old female being treated with 250 mg daily of gefitinib daily for lung adenocarcinoma and bone metastasis, who experienced moderate to severe bilateral sensorineural hearing loss after 4 months of initial treatment. The patient discontinued treatment after symptoms initially arose, which resulted in partial return of auditory function, but was restarted after her cancer-related symptoms persisted. This resumption of gefitinib treatment led to worsening deafness that persisted until the patient passed away after continuous treatment (Zhu et al., 2023).

Sunitinib is a TKI that has been approved for several clinical applications, including treatment of retinal cell carcinoma in 2006 (Motzer et al., 2017), GIST in 2006 (Goodman et al., 2007) and pancreas neuroendocrine tumors (NET) in 2011 (Blumenthal et al., 2012). It is known to have a wide range of targets such as PDGFR-α and β, VEGFR-1,2, and 3, CSF1R, c-kit, RET, and FLT3 tyrosine kinases (Mena et al., 2010). To date, there has been only one case described in the literature of massive right side SNHL following treatment with 37.5 mg of sunitinib daily. The hearing loss was observed 15 days after initial treatment, along with other aural vestibular symptoms, despite a comprehensive neurotologic evaluation reported to be within normal limits. The sunitinib treatment was discontinued, and high-dose corticosteroids were used as an attempt at recovering hearing without success (Dekeister et al., 2016).

Osimertinib is another EGFR TKI that was approved for use treating NSCLC in 2015 (Greig, 2016). Literature review only revealed one case of potential ototoxicity. A 71-year-old male with advanced lung adenocarcinoma and treated with 80 mg per day of Osimertinib. This patient experienced tinnitus and progressive bilateral hearing loss 6 months after initial treatment. After formal audiologic evaluation, a moderate to severe bilateral SNHL, the patient continued the treatment despite appropriate counseling of the risks and developed bilateral severe to profound sensorineural hearing loss detected 1 year after. Hearing rehabilitation was recommended with some improvement in his quality of life (Lim et al., 2022).

When evaluating the reported protective effects of EGFR inhibition against noise-induced hearing loss, it is important to account for dose-, duration-, and patient-specific factors. Low-dose, short-term EGFR inhibition may exert beneficial effects via anti-inflammatory and anti-apoptotic pathways (Vijayakumar et al., 2024). In contrast, high-dose or prolonged use, particularly in oncology settings, may disrupt normal cochlear homeostasis. Notably, many patients receiving EGFR inhibitors have also been exposed to established ototoxic agents such as platinum-based chemotherapy and radiation, complicating attribution (Zhu et al., 2023). A synergistic or compounding effect is possible, though in some cases, the observed association between EGFR inhibition and hearing loss may be incidental rather than causative.

#### Proteasome inhibitors: bortezomib, carfilzomib, marizomib

Bortezomib is a proteasome inhibitor that has been approved for the treatment of multiple myeloma (MM) since 2003 (Kane et al., 2003) and mantle cell lymphoma (MCL) in 2014 (Raedler, 2015). There have been three reported cases of ototoxic symptoms that arose in association with bortezomib treatment. The standard treatment across cases is 8 cycles of 2 weeks of 1.3 mg/m^2^ intravenously twice per week, followed by a week of no treatment. The first case was of a 62-year-old male diagnosed with MM who stopped bortezomib treatment after experiencing severe bilateral SNHL after 4 cycles, and one even after a dose reduction to 1.0 mg/m^2^ after the third cycle (Engelhardt et al., 2005). The second case was a 42-year-old female diagnosed with stage III MM treated with intravenous bortezomib. After her second cycle, audiologic testing confirmed left sensorineural hearing loss, stopping as a result. Patient died 8 months after the cessation of treatment with no improvement in hearing loss (Chim and Wong, 2008). Additionally, a 56-year-old female with MM experienced bilateral sensorineural hearing loss following the third dose of bortezomib, which was later discontinued. Audiologic testing performed 16 weeks after the discontinuation of therapy showed no hearing improvement (Anoop et al., 2016). Lastly, a 67-year-old male with Waldenstrom macroglobulinemia reported asymmetrical sensorineural hearing loss after the third dosage of bortezomib, with profound loss in the right ear and moderate to severe loss in the left, requiring cochlear implantation surgery with good postoperative outcomes (Fitzsimons et al., 2024).

The etiology of bortezomib-induced hearing loss is unclear, but proteasome inhibitors have been associated with peroxisome dysfunction, which can lead to auditory hair cell death. Table 2 provides a summary of the discussed small molecules.

**TABLE 2:** Summary of small molecules and inner ear effects reported.

Small molecule drug	Drug classification	Hearing effects reported	References
Imatinib	TKI	- Symmetrical/Asymmetrical Moderate to Severe Bilateral SNHL	Attili et al. (2008) Lin et al. (2012) Lim et al. (2022) Wasif et al. (2016) Gupta et al. (2017) Flynn Gerriets (2025)
Erlotinib	TKI	- Severe Asymmetrical Bilateral SNHL	Koutras et al. (2008)
Gefitinib	TKI	- Moderate to Severe Bilateral SNHL	Zhu et al. (2023)
Sunitinib	TKI	- Massive Right-Side Hearing Loss	Dekeister et al. (2016)
Osimertinib	TKI	- Moderate to Severe Bilateral SNHL	Lim et al. (2022)
Bortezomib	Proteasome Inhibitor	- Symmetrical/Asymmetrical Moderate to Severe Bilateral SNHL	Engelhardt et al. (2005) Chim Wong (2008) Wong et al. (2008) Anoop et al. (2016) Fitzsimmons et al. (2024)

#### Screening and follow-up

Across all case reports and systematic reviews, time-to-follow-up was highly variable after hearing loss. Audiometric data was inconsistently recorded; for most mAbs, in which only one to two cases were described, where patients reported subjective hearing loss, formal audiometric testing was never performed. Furthermore, no identified studies reported regular audiometric screening during treatment. Testing was most frequently performed after onset of symptoms and following treatment (most commonly with intratympanic steroid injections), to assess recovery.

The American Speech-Language-Hearing Association (ASHA) guidelines define medication-induced ototoxicity as a 20 dB threshold decrease at any frequency, 10-dB decrease at any two consecutive frequencies, or no response at three consecutive frequencies where responses were previously present (Konrad-Martin et al., 2005). However, these criteria only apply if the patient has had a prior hearing test performed. In cases without prior testing, the physician must rely in the patient’s subjective complaint, physical exam findings, and most recent audiogram in association to recent history of exposure to any of the above-mentioned therapies without any other possible conflicting risks factors (e.g. recent history of head trauma, radiation therapy, prior chemotherapy with known ototoxic drugs, use of long-term intravenous antibiotics, recent viral infections, amongst others). Therefore, a comprehensive head and neck evaluation by an otolaryngologist with or without subspecialty in otology-neurotology and an audiometric assessment performed by an audiologist is important before the initiation of therapy, during treatment, especially if the patient experiences new or worsening audiovestibular symptoms, and after completion of treatment.

There are two main classes of drugs which merit regular audiometric evaluation for ototoxicity: aminoglycoside antibiotics, and platinum-based chemotherapies (Schlatcter et al., 2023). Other drugs also carry known, albeit lower, risk of ototoxicity, including salicylates and loop diuretics (Rybak and Ramkumar, 2007). According to the American Academy of Audiology, current best practice for ototoxicity screening in known offending agents emphasizes early detection of hearing loss with use of high-frequency audiometry (HFA) and otoacoustic emission (OAE) testing (Durrant et al., 2009). However, in cases where the ototoxic profile of a drug-such as many immunotherapies-is unclear, screening usually consists of conventional audiometry (0.25–8 kHz) supplemented by validated questionnaires for tinnitus (Newman et al., 1998) and dizziness (Campbell and Durrant, 1993) as needed. Test selection should balance sensitivity and patient burden, with tools like auditory brainstem response (ABR) reserved for patients whose behavioral response is inadequate and cannot reliably answer survey instrument tools. There is significant potential for modification of screening and monitoring of possible ototoxic agents; testing can and should be adapted based on the specific ways a drug is known or hypothesized to affect the inner ear (Campbell and Le Prell, 2018). Since this is a complex medical decision-making process, all patients should be evaluated by a multidisciplinary team involving an otolaryngologist and an audiologist.

A significant number of case reports establish connections between the discussed pharmacologic agents and hearing loss based solely on patients’ subjective accounts, rather than utilizing objective audiometric assessments. Even in cases where formal audiometry is performed after hearing loss occurs, it can be difficult to determine the exact ototoxic effect when hearing baselines are unknown. The strongest association exists in cases in which audiometric testing was recorded as a baseline prior to treatment initiation. The most performed diagnostic test was pure tone averages, while others such as word recognition scores (WRS) or speech reception threshold (SRT), were also used. The ASHA recommends full audiologic workup for known ototoxic agents, including pure-tone audiometry, including extended high-frequency audiometry (9000–20,000 Hz), speech audiometry (SRT and WRS) and OAE studies. Other existing objective tests include tympanometry, acoustic reflex testing, ABR, and electrocochleography that are used to evaluate middle ear function, auditory nerve integrity, and cochlear responses, respectively. No protocols yet exist for the routine monitoring of the immunotherapies discussed in this review, including which specific diagnostic tests to include in initial screening, or at which intervals to assess throughout the duration of treatment. Even for well-established ototoxic agents such as aminoglycosides and platinum-based agents, routine hearing screening is not consistently implemented at scale, and standardized monitoring protocols (Lord, 2019) are broadly underutilized and variably applied across institutions. This lack of consistency is usually related to lack of awareness of treating teams and inappropriate patient counseling.

There is high clinical benefit of establishing baseline screening prior to initiation of immunotherapy; one major reason is that assessment of causality would be strengthened, as objective changes could be tracked over time after drug initiation. Routine screening allows early detection and intervention in cases of subclinical hearing loss. While the overall incidence of immunotherapeutic ototoxicity is low, screening has a general benefit to patient care by facilitating early detection of age-related or multifactorial hearing loss, establishing a reference point for future comparisons, and improving provider confidence in managing auditory symptoms that arise during treatment. It also supports more informed shared decision-making, especially in complex patients where multiple etiologies for hearing changes may coexist.

## Cost-benefit analysis

Cost-Benefit Analysis has been conducted to compare hearing screening schedules in the general adult population with special attention to quality-adjusted life-years (QALY) and age at which screening begins. By U.S. standards, screening was deemed to be cost-effective for adults beginning at 55 years of age at a 5-year interval. In the model used, screening was most cost-effective at older ages (starting at age 65 or 75 versus age 55), aligning with the fact that age is the most common predictor of hearing loss. Routine screening also led to increased utilization of hearing aids (Borre et al., 2023). However, this evidence may not directly apply to patients receiving immunotherapy. One possible correlation for this study’s population concerning cost limitation is to screen selectively based on risk factors. Factors that would include a patient for screening may include prior otologic injury, autoimmune conditions, or history of non-otologic systemic adverse reaction to immunotherapy (Hsu et al., 2022).

## Limitations

Several limitations of the study and its source material warrant discussion. This review includes studies with varying levels of evidence and a limited capacity to establish causation; much of the data is drawn from database analyses and case reports with small or inconsistent sample sizes. Many patients receiving these drugs are medically complex, often with multiple comorbidities or concurrent insults that could contribute to ototoxicity, complicating efforts to attribute hearing loss directly to the drug in some cases. Due to the characteristics of the included studies, it is also difficult or impossible to determine whether hearing loss occurred due to immunotherapeutic use or other known ototoxic treatment, such as cisplatin or exposure to radiation therapy. Therefore, those patients with history of prior or concurrent platinum-based therapies, any causal conclusions are speculative. Additionally, baseline hearing assessments were rarely conducted, and post-injury testing and treatment were inconsistently performed and variably reported. These limitations make it difficult to identify clear trends in the timing of otologic injury or response to treatment. The importance of our review is the demonstration of inconsistent availability of objective hearing measurements both pre–and post-injury across the literature. The author’s goal is to raise awareness and promote the creation of protocols and/or guidelines that encourage baseline assessments and standardized workup of patient undergoing treatment with any of the above-mentioned drugs.

### Final remarks

Cases of hearing loss associated to targeted therapies such as the discussed in this manuscript are rare compared to conventional chemotherapy, especially platinum-based antineoplastic drugs. However, it is recommended that every patient receiving chemotherapy, including targeted therapy, be evaluated by a multidisciplinary team including an otolaryngologist and an audiologist for a comprehensive neurotologic examination and audiometric testing before the beginning of therapy. Patients should also be counseled regarding the potential ototoxic side effects of the medications and instructed to reach out to the otolaryngologic specialist if experiencing sudden changes of aural symptoms (subjective decreased hearing, new onset of tinnitus and/or ear clogged sensation) that do not improve within 72 h. Otherwise, regular hearing screening should be performed every 6–12 months.
